# Variation in neophobia among cliff swallows at different colonies

**DOI:** 10.1371/journal.pone.0226886

**Published:** 2019-12-23

**Authors:** Stacey L. Hannebaum, Gigi S. Wagnon, Charles R. Brown

**Affiliations:** Department of Biological Sciences, University of Tulsa, Tulsa, Oklahoma, United States of America; Universidade de São paulo, BRAZIL

## Abstract

Animal groups often represent nonrandom subsets of individuals, and increasing evidence indicates that individuals may sort among groups based on their personalities. The size of a group can predict its personality composition in some species due to differential suitability of a personality for groups of certain sizes, and the group itself may function more effectively if particular personality types are present. We quantified cliff swallow (*Petrochelidon pyrrhonota*) behavioral measures using linear and generalized linear mixed models to identify whether they: (1) varied among individuals within colonies and among colonies, (2) were related to reproductive success, and (3) predicted levels of parental care. Significant among-individual and among-colony site variation in a cliff swallow’s latency to enter its nest when presented with a novel stimulus was revealed. We also found significant among-individual variation in the number of attacks directed toward a novel stimulus at the nest and in the response to broadcast of a cliff swallow alarm call recording, but among site variation in these measures was not significant. We did not find evidence for behavioral syndromes linking the personalities measured. Differences among individuals in latency to enter the nest and the number of attacks were not significantly related to reproductive success or to the extent to which birds fed their nestlings. However, extent of nestling feeding was significantly predicted by the number of mist net captures. The limited evidence in general of systematic variation in the behavior we measured among cliff swallow colonies may reflect the different and sometimes opposing selection pressures on behavior in different social environments. Future work should perhaps examine variation in other behavioral traits, such as foraging, in cliff swallow colonies of different sizes.

## Introduction

Animal groups often represent nonrandom subsets of individuals, with the composition of groups known to vary with characteristics such as age, experience, life-history investment, morphology, cognitive abilities, parasite resistance, and physiology of individuals [1–3]. Groups also can differ in the behavioral profiles of their constituent members [4–10], and increasing evidence indicates that individuals may sort among groups based on their personalities [3, 11, 12]. Size of a group can predict behavioral variation in some species [11–14]. Individuals with particular personalities may be better suited for groups of certain sizes [6, 11, 15, 16], and the group itself may function more effectively if particular personality types are present [17–19]. Systematic behavioral variation among groups could be important in explaining fitness differences among individuals and among groups, and may be key in understanding why both the size and phenotypic composition of animal groups vary in the first place [3, 20].

An individual’s propensity to take risks (boldness; [21, 22]) and how an individual reacts to a novel situation (exploration; [22]) are personality traits that offer different advantages depending on group size. For example, whereas individuals in large groups may have greater chances of avoiding a predator, compared to individuals alone or in small groups [23–27], the increased number of individuals assessing threats in a large group means the odds of misclassifying a threat and broadcasting a false alarm increases [28, 29]. Thus, bold individuals that ignore some alarm cues would likely benefit more in larger groups than would shy individuals that flee with every false alarm and waste time and energy [30–32].

Breeding colonies of animals offer many opportunities to study behavioral variation and its consequences. For example, in colonial spiders, individuals with different levels of aggressiveness benefit differently depending on the size of the colony they inhabit [6, 11]. Specifically, aggressive individuals benefit most by being in small colonies where there are fewer opportunities to waste time and energy fighting with neighbors, whereas shy individuals can occupy large colonies without the costs of constantly fighting with many conspecifics there [6].

Although behavioral variation in colonial spiders has been studied extensively, little is known about personality of colonial vertebrates. In one of the few studies on a colonial bird, Dardenne et al. [12] found that larger barn swallow (*Hirundo rustica*) colonies contained individuals with higher levels of neophobia, a term that characterizes hesitation and avoidance behavior in reaction to a novel stimulus and may also affect exploration [22, 33–35].

Cliff swallows (*Petrochelidon pyrrhonota*) breed in colonies that vary widely in size [36]. What maintains colony-size variation in cliff swallows (and other species) is an unresolved problem, but one possibility is that different colony sizes reflect sorting of individuals with different personalities into the social environment in which they do best [3, 14, 15]. Although several studies have investigated various types of phenotypic sorting among group sizes in cliff swallows [37–41], little is known about whether colonies consistently vary in the personalities of group members [14].

In this study, we quantified cliff swallow behavioral measures to identify whether they: (1) varied among individuals within colonies and among colonies, (2) were related to reproductive success, and (3) predicted levels of parental care, a possible mechanism contributing to variation in fitness [42]. We focused on four measures of behavior related to neophobia and risk-taking: (1) latency to enter a nest bearing a novel stimulus, (2) the number of attacks towards a novel stimulus at the nest, (3) the number of captures in a mist net placed at the colony, and (4) the responses to playbacks of conspecific alarm calls. The intent was to use the results to better understand how the phenotypic composition of cliff swallow colonies varies and if any such variation could reflect advantages of certain colony sizes for particular behavioral phenotypes.

## Materials and methods

### Study area and animal

This study was conducted near the Cedar Point Biological Station (41.2097°N, 101.6480°W) in Keith County, southwestern Nebraska, USA. Cliff swallows have been studied in this area and the surrounding counties (Morrill, Garden, Deuel, and Lincoln) since 1982 [37]. Birds arrive in the study area beginning in late April and form colonies for the breeding season, which extends to mid-July. Cliff swallows build gourd-shaped mud nests mostly on the sides of bridges or inside box-shaped culverts under roads and railroad tracks, but a few still use horizontal overhangs on the sides of steep cliffs [36]. Approximately 220 colony sites exist within the study area, with about a third of those not used in a given year. The size of a colony varies from 2 to 6000 nests ($\bar{X}\pm \text{SE}=404\pm 13$, *N* = 2318 colonies); some birds also nest solitarily. Colonies were defined as birds from groups of nests that interacted at least occasionally in defense against predators or by sharing information on the whereabouts of food [37]. Colony size refers to the maximum number of active nests at a site in a season, with an active nest defined as one in which at least one egg was laid. Direct counts of all active nests (from inspecting nest contents) or estimation based both on nest counts of portions of a colony site and on the number of birds present at a site were used to determine colony size [36, 37]. For further description of the study area, see [37].

**Fig 1:**
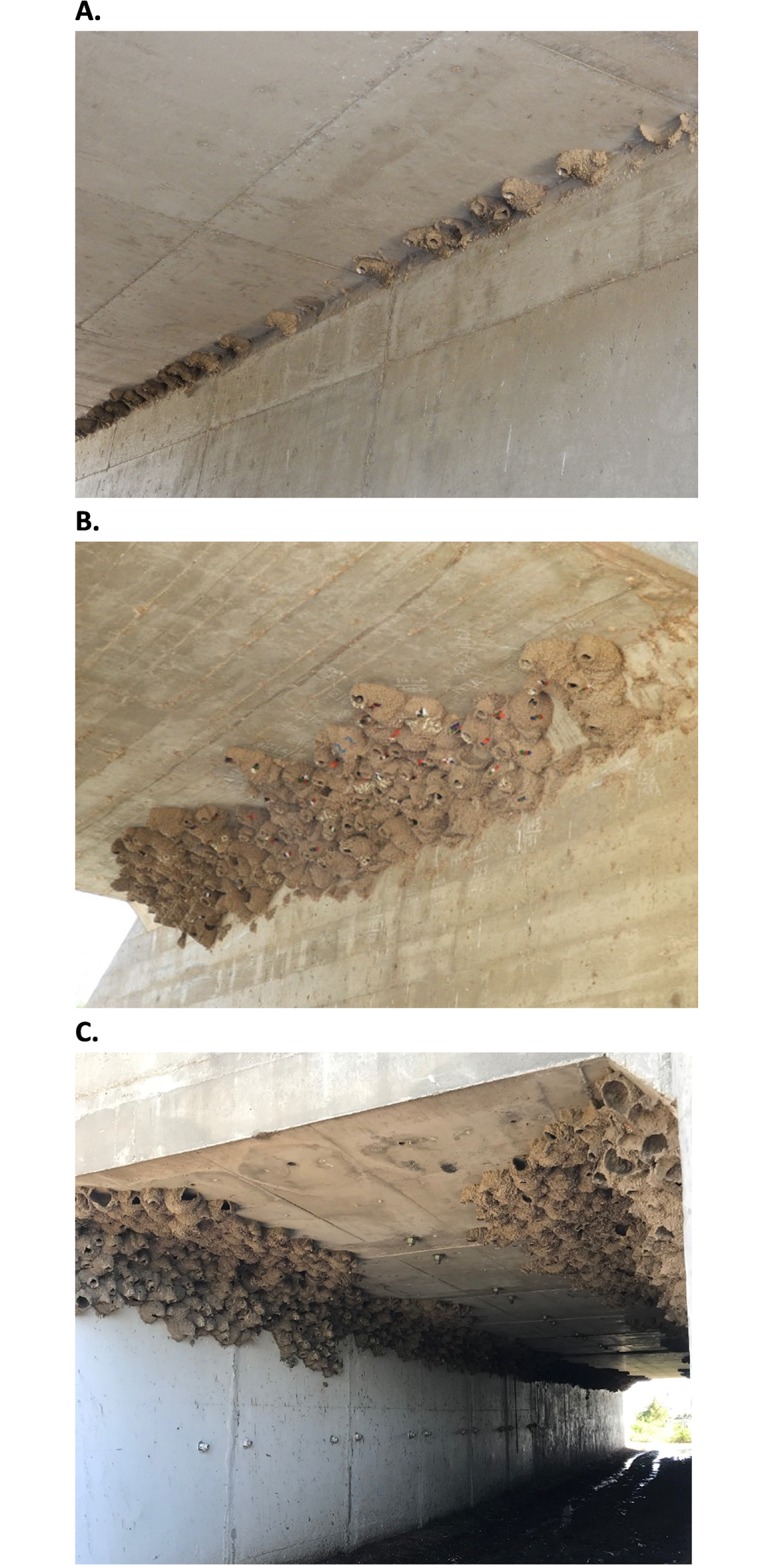
Comparison of nest density and extent of clustering at three colony sites of different colony sizes. (A) McDougals site versus the (B) CR-1 and (C) Junkyard sites.

We used six colonies that occurred at different times at three colony sites named McDougals (41.311°N, -102.002°W), CR-1 (41.209°N, -101.635°W), and Junkyard (41.252°N, -101.619°W). The sites were situated on public right-of-way and required no landowner permission to access. The McDougals and Junkyard sites contained active colonies in both years of the study whereas the CR-1 site was only active in 2017 (Table 1). In 2018, McDougals had two separate colonies defined by time (early and late). The birds in these two colonies had little temporal overlap (average egg-laying date was 20 May for the early colony and 1 July for the late one), and hence were considered functionally distinct colonies. The sizes of colonies at each site were relatively consistent within and between years (Table 1): colonies at McDougals were small, colonies at Junkyard were large, and the colony at CR-1 was medium in size. The colony sites also differed in nest density and extent of clustering as a result of colony size and the size of the physical nesting substrate (Fig 1).

**Table 1:** Observation effort and mean values for cliff swallow behavioral tests, parental food deliveries, and annual reproductive success at six colonies over two years.

	Descriptive statistics	Colony site					
		2017 McDougals	2018 McDougals (early)	2018 McDougals (late)^a^	2018 CR1	2017 Junkyard	2018 Junkyard
	colony size^b^	44	53	75	525	1815	1920
Neophobia tests^c^	*n*_ind._^d^	27	28	15	41	24	25
	*n*_obs._^e^	101	84	45	144	92	67
Latency to enter nest^f^	mean (± SD)	201.7 (113.31)	179.3 (120.78)	143.1 (120.93)	93.1 (109.14)	89.5 (111.12)	134.7 (125.52)
Number of attacks	mean (± SD), range	1.7 (2.61), 0–13	2.3 (3.30), 0–21	1.6 (2.26), 0–10	1.0 (1.48), 0–8	2.2 (3.01), 0–13	1.7 (2.37), 0–13
Number of captures	mean (± SD), range	1.8 (1.05), 1–5	1.3 (0.59), 1–3	1.2 (0.41), 1–2	1.2 (0.46), 1–3	1.1 (0.28), 1–2	1.0 (0.20), 1–2
Alarm call response tests	*n*_ind._^d^	28	40	19	48	56	35
	*n*_obs._^e^	140	116	53	144	295	102
	*% n*_stayed_^g^	85.0	82.8	79.2	79.2	80.0	79.4
	*% n*_fled_^h^	15.0	17.2	20.8	20.8	20.0	20.6
Parental food delivery observations	*n*_nests_^i^	16	18		32	23	23
	*n*_obs._^e^	213	85		134	342	89
	mean (± SD), range	14.6 (6.72), 2–42	15.4 (4.74), 2–31		11.3 (4.90), 1–26	11.0 (6.94), 0–42	8.8 (4.43), 1–22
Reproductive success	mean (± SD), range	3.0 (1.29), 0–4	3.8 (0.84), 2–5		2.9 (0.92), 0–4	2.5 (1.02), 0–4	2.5 (1.12), 0–4

^a^ Measures of parental care and reproductive success were not obtainable for this colony due to time constraints.

^b^ The number of active nests

^c^ Includes both Latency to enter nest and Number of attacks

^d^ The number of individuals observed

^e^ The number of observations conducted

^f^ Range was 0–300 for all colonies

^g^ The percentage of individuals that stayed in the nest during an alarm call broadcast

^h^ The percentage of individuals that fled from the nest during an alarm call broadcast

^i^ The number of nests observed

### General field methods

In 2017 and 2018, cliff swallows at selected colony sites were captured in mist nets as they exited their nests (see [37] for detailed methods). Mist-netting of each colony occurred during egg-laying and within a single day, except at the 2017 McDougals colony at which different portions of the colony were netted over a consecutive two-day period (to increase sample size) with different birds captured each day. The number of times an individual was captured during the single day of netting was recorded as “Number of captures.” Birds caught were sexed, weighed, given a unique United States Geological Survey band if not already banded, color-marked for later identification in the field, and released. The white forehead patch of each bird was color-marked with a unique 3-color stripe combination using permanent, non-toxic Sharpie^®^ markers; the forehead patch is highly visible when a cliff swallow is at its nest [37]. A total of 646 cliff swallows were color-marked and, of those, 226 were identified to specific nests and later observed.

Each colony was observed the day following mist-netting to determine at which nests color-marked birds resided. Repeated nest visits and nest defense were indicators of nest residency. At CR-1, which had complex nest stacking, 3-stripe color combination codes were painted on the nests matching the color-mark pattern of the resident bird(s) to facilitate observation. Behavioral tests were conducted primarily by the same two observers (SLH, GSW) in both years. Observers sat in chairs or on the ground as close to the nests as possible (typically within a meter of the culvert entrance) without noticeably influencing the behavior of the birds, and cliff swallows at all sites seemed to rapidly habituate to the presence of stationary humans. Three individuals observed at McDougals in 2017 were observed again at McDougals in 2018. The behavioral measures of these individuals were not consistent between years; therefore, these birds were treated as different individuals each year. Individuals known to be resident in both 2018 McDougals colonies were observed only during the early or late colony but not during both.

The nests used in this study were lightly misted with a dilute solution (1:170 parts water) of naled (Dibrom 8) applied every 7–10 days to remove ectoparasitic swallow bugs (Hemiptera: Cimicidae: *Cimex vicarius*) as part of other research [37, 43, 44]. Naled is an organophosphate insecticide that is highly effective against swallow bugs [44, 45]. Swallow bug infestations vary among nests and colonies [37], and reduced reproductive success has been linked to parasitism [37]. We removed swallow bugs to reduce the likelihood of nest failure before observations were completed.

### Alarm call response test

This test measured individual response to a playback of a cliff swallow alarm call recording. At the start of each trial, the “before” status of typically 3 to 5 focal birds was recorded as either an individual being in its nest and visible to the observer, or in its nest and not visible to the observer. Once the before status was determined, a 15-sec audio clip of alarm-calling cliff swallows was broadcast from an iPhone, set at full volume, to the colony through a Dennis Kirk Electronic Wildlife Caller, set at mid-volume, in 2017 or an Ultimate Ears Wonderboom Portable Bluetooth Speaker, set at mid-volume, in 2018. Six different audio clips were used between the two years (see S1 File). In 2017, one audio clip was used exclusively for several days until the birds no longer reacted to the alarm call; then a different audio clip was played. Once birds stopped reacting to the second audio clip, a third audio clip was used until behavioral testing was completed. In 2018, three different audio clips were used in a fixed sequence, rotating with each alarm call broadcast. Habituation to alarm calls was less obvious in 2018. Once the audio clip finished playing, the after status of each focal bird was recorded as the individual either remaining in its nest or having flown from the nest. These “Alarm call response” trials were performed at least 20 minutes apart in 2018, but there was no such designated time separation during 2017. Mean time between trials was 40.0 min (SD = ± 47.24, range = 1–268, *N* = 192 trials) for 2017 and 53.8 min (SD = ± 47.98, range = 20–309, *N* = 310 trials) for 2018. Most birds were observed at least 3 times ($\bar{X}\pm \text{SD}=3.8\pm 1.45$, range = 2–8, *N* = 226 birds) over the course of the study.

### Neophobia test

**Fig 2:**
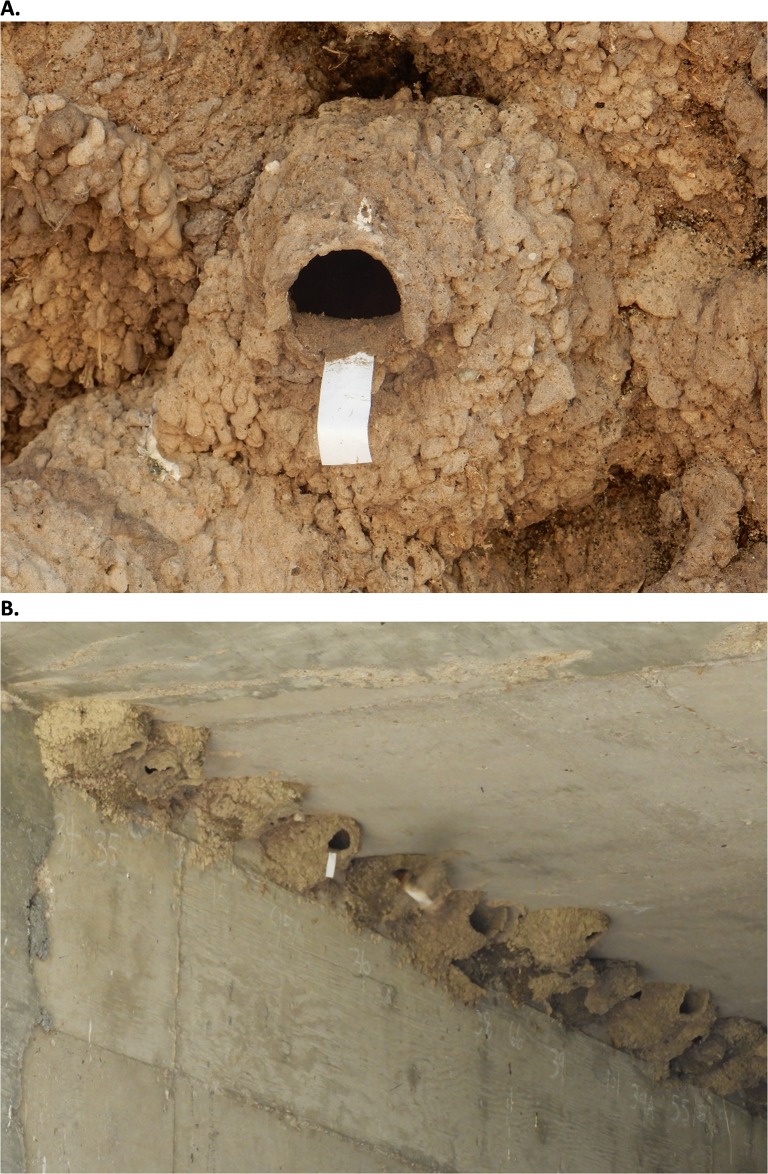
Marking tape (novel object) placement on a cliff swallow nest as used in neophobia trials measuring latency to enter nest and number of attacks. (A) Marking tape on a nest and (B) a cliff swallow hovering to the right of its nest.

The second behavioral test measured two behaviors related to neophobia. For this test, a single nest (1–2 focal birds depending on whether both residents were color-marked) was observed during a trial. Once all color-marked residents were inside the nest, the observer walked toward the nest until the residents flushed from the nest and out of the culvert. The observer then immediately attached a 1.5- x 5-cm strip of white marking tape to the middle of the lower lip of the nest entrance using wet mud, allowing ~4 cm of the tape to dangle freely (Fig 2), then returned to the observation location. A timer was started for each focal color-marked bird when the observer first saw the bird return to the culvert and was stopped when the focal bird entered the nest or when 300 sec (5 min) had passed without nest entry. This time was noted as “Latency to enter nest.” During this time, birds often repeatedly hovered in front of the nest while observing the marking tape, then flew out of the culvert, and returned to hover in front of the nest again. Birds occasionally attacked the marking tape while hovering. A quick burst toward or actual physical contact with the marking tape defined an attack, and the number of attacks that occurred during the Latency to enter nest were recorded as “Number of attacks.” Attacks sometimes occurred in rapid succession during one hover event in front of the nest, but attacks could also be separated by time spent outside of the culvert. The marking tape was removed from the nest once the trial was finished. To minimize habituation to the marking tape, the birds at a given nest experienced a trial only once in a day and never experienced trials on consecutive days. Most birds underwent at least 3 neophobia trials ($\bar{X}\pm \text{SD}=3.3\pm 0.61$, range = 2–4, *N* = 160 birds) over the course of the study.

### Measuring reproductive success and parental food deliveries

We monitored the content of nests by directing a flashlight beam onto a dental mirror inserted through a nest’s tubular opening. Nests were checked every 4 days to allow for estimation of laying and hatching dates and to monitor the age and number of nestlings present (see [37] for hatch date estimation methods). At 17 days after the estimated hatching date, the number of nestlings surviving in each nest was determined and used as a relative estimate of reproductive success for each focal bird or nest. Cliff swallows typically fledge at 23 to 26 days of age [46]; we chose to use survival to 17 days as a proxy for successful fledging and to minimize the likelihood of premature fledging that can happen after day 17 if nests are disturbed.

The number of parental food deliveries per hour was recorded for nests of color-marked birds that contained nestlings. We counted only visits in which food was brought to the nest, as evidenced by observation of direct transfer to the nestlings’ mouths or by observation of parental tail-pumping, a behavior characteristic of parents as they place food in the nestlings’ mouths. Parental food deliveries were recorded for each nest rather than for each individual because of the difficulty in confidently identifying the individual during this activity and because color-marks were significantly faded by this time. Food-delivery observations were conducted primarily by the same observer (SLH) both years. Typically, 5 to 20 nests were monitored during an hour. All nests were observed at least twice and most nests were observed at least 4 times ($\bar{X}\pm \text{SD}=7.7\pm 5.08$, range = 2–18, *N* = 112 nests; Table 1). Age of nestlings ranged from 1 to 29 days ($\bar{X}\pm \text{SD}=14.4\pm 5.24$, *N* = 863 observations) during food delivery observations.

Because cliff swallow foraging (and possibly the other behavior we observed) is strongly dependent on local weather conditions [37], hourly weather covariates (temperature measured as °C, wind speed measured as m/sec, and extent of sunshine measured as solar radiation in watts/m^2^) from the study area were included in our analyses. These variables, recorded to the nearest hour of when we observed a given nest or performed a given behavioral trial, were extracted from the High Plains Regional Climate Center’s Automated Weather Data Network (http://awdn.unl.edu/classic/home.cgi). All weather data were collected from the Keystone (BETA) station (41.202° N, -101.645° W; approximately 1–32 km away from colony sites) except for dates prior to 13 June in 2018 when this station was offline and data from Big Springs (41.159° N, -101.995° W; approximately 17–33 km from colony sites) were used.

### Ethical note

Birds were captured, handled, banded, and released under authority of the Bird Banding Laboratory of the United States Geological Survey (permit 20948) and a Scientific Permit from the Nebraska Game and Parks Commission (permit 1033). All animal use was approved by the University of Tulsa Institutional Animal Care and Use Committee (TU-0047, TU-0048).

### Analysis of among-individual and among-colony site repeatability

We used linear and generalized linear mixed models to evaluate whether there was significant variation in behavior among individuals and among colony sites by including the identity of each individual (Bird ID) and the three colony sites (Site ID) as random effects [47, 48]. We chose to model the colony site as a random effect rather than colony size because colony size was relatively consistent at each site within and across years (Table 1). Latency to enter nest was converted to a proportion ((*x* + 1)/301–0.001) and logit-transformed to fulfill linear modeling assumptions [49]. Number of attacks and Number of captures were skewed toward minimal and low values, characteristic of count data; thus, these were modelled with a Poisson distribution. Because we only had one measure of the Number of captures per individual, only Site ID was used as a random effect for this model. Alarm call response (0 or 1) was modeled with a binomial distribution.

We included a set of biologically reasonable independent predictor variables as fixed effects in each model to control for environmental and demographic factors that could influence cliff swallow behavior. Fixed effects included in the analyses of Latency to enter nest and Number of attacks were sex, neophobia test rank order (to control for habituation), days since first egg was laid (to control for parental investment, ranging from -5 to 31, indicating some trials were done before eggs were laid and others were done after eggs had hatched), temperature, wind speed, and extent of sunshine. Latency to return to the nest (untransformed) was also included as a predictor of Number of attacks. Fixed effects included in the analysis of Alarm call response were sex, alarm broadcast rank order (to control for habituation, the number of times a given alarm recording had been used at the colony at a given point in time), status before alarm (visible or not visible), days since first egg was laid (ranging from -10 to 29), temperature, wind speed, and extent of sunshine. Sex was included as the sole predictor variable for Number of captures. All predictor variables were modeled as continuous linear coefficients except for sex, neophobia test trial number, and status before alarm, which were modeled as categorical. Interactions among linear covariates were not modelled due to insufficient sample size. All linear predictor variables were standardized ($\left[\bar{X}-x\right]/\text{SD}$). We used the function lmer (Latency to enter nest) or glmer (Number of attacks, Alarm call response, Number of captures) from the R package “lme4” [50] to fit the mixed models (see S2 File).

To determine the extent of variation in each behavioral measure that can be explained by the individual or site, we calculated the repeatability, *R*, which is the proportion of variance across individuals (Bird ID) or sites (Site ID) divided by the total variance. Behaviors that are repeatable within an individual define a personality [22, 51, 52]. Adjusted repeatabilities, accounting for fixed effects, were calculated following Nakagawa and Schielzeth [53] using the function rpt from the R package “rptR” [54]. In each calculation, a behavioral measure was used as the response variable and Bird ID and Site ID as group variables. Given the non-normal distribution of Number of attacks, Number of captures, and Alarm call response, we specified Poisson GLMMs for the former two behavioral measures and a Binary GLMM for the latter, and estimated repeatability on the latent (log-link for Number of attacks and Number of captures and logit-link for Alarm call response) scale [53]. The number of bootstrap iterations was set at 1000 (see S2 File). Statistical significance of the repeatability was tested using the likelihood ratio test (LRT) implemented in rptR.

### Analysis of behavioral correlations

To test for correlations between behavioral measures, the presence of which indicates that the behavioral tests measure the same personality trait or represent a behavioral syndrome [5, 55], we used bivariate generalized linear mixed models fit using the MCMCglmm function in the R package “MCMCglmm” [56]. Bivariate models estimate the covariance between two response variables, allowing for direct correlation estimates with valid measures of uncertainty [57, 58]. Models were fit to test for correlations between all possible variable combinations except Alarm call response. Alarm call response was not included because correlations cannot be calculated for binary data. The same fixed effects listed above were specified for Latency to enter nest, Number of attacks, and Number of captures (see S3 File). Bird ID and Site ID were included as random effects and were set with an unstructured (‘us’) G-structure. We also set the covariance matrix as unstructured (‘us’). The family argument was specified as ‘Gaussian’ for Latency to enter nest and as ‘Poisson’ for Number of attacks and Number of captures. Because we only had one measure of Number of captures per individual, the variance of Number of captures was fixed at a low value (0.0001) in the prior. We used inverse-gamma priors throughout and ran all models for 750000 iterations, with a burn-in of 50000 and a thinning interval of 175 (see S3 File). Successive samples from the posterior distribution had low autocorrelations (*r* < 0.01). We calculated both point estimates and credible intervals for the covariates between each pair of behavioral measures using the posterior modes and highest posterior density (HPD) intervals estimated in each bivariate model. HPD intervals not overlapping zero indicate significant correlation.

### Analysis of correlations between behavior and reproductive success

We used bivariate generalized linear mixed models fit using the “MCMCglmm” package to test for correlations between behavioral measures and Reproductive success. Bivariate models of Latency to enter nest and Reproductive success and Number of attacks and Reproductive success were fit as described above (see S3 File). As in Number of captures, we only had one measure of Reproductive success per individual, so the variance of Reproductive success was fixed at a low value (0.0001) in the prior. Sex, first egg lay date, and clutch size were included as fixed effects for Reproductive success. Again, HPD intervals were calculated from each model to determine whether the correlation was significant.

### Analysis of behavioral predictors of parental care

We assessed whether behavioral traits were associated with the frequency of parental food deliveries by modeling the number of food deliveries to a nest per hour as the dependent variable in a linear mixed model with date, number of nestlings, nestling age, temperature, wind speed, extent of sunshine, and three nest-level behavioral scores (Latency to enter nest, Number of attacks, and Number of captures) as fixed effects. Because feeding frequency increases with nestling age until 10 days of age, plateaus until 17 days of age, and then decreases [37], we modeled nestling age as a quadratic coefficient to account for this curvilinear relationship. Nest ID and Site ID were included as random effects.

We fit univariate mixed models of Latency to enter nest and Number of attacks using the same distribution families and fixed effects used in previous univariate and bivariate models and Nest ID as a random effect so that we could calculate conditional modes (best linear unbiased predictors, BLUPs) of the Nest ID random effect from each mixed model. These BLUPs predict the random effect term independent of potential confounding factors in the mixed model [59]. We extracted the BLUPs for use as a nest’s relative score of Latency to enter nest and Number of attacks [46, 60, 61] rather than calculating a mean score for each nest, which can be highly influenced by extreme values and problematic when data exhibit a Poisson distribution [59]. However, we acknowledge that the use of BLUPS, which are predicted to contain high error, in secondary analyses can lead to anti-conservative confidence intervals [56]. Because we only had two values for Number of captures per nest (unmarked mates of observed marked birds were given a Number of captures value of zero), we calculated a mean score of Number of captures for each nest to use in our model.

The parental food delivery mixed model was performed in SAS [62] and all other statistical tests were performed in R 3.5.2 [63]. Because we had a biological rationale for all of the independent predictor variables used [37, 61, 64], we did no model selection for these analyses.

## Results

We obtained 533 neophobia behavioral observations for 160 color-marked cliff swallows, 850 alarm call response behavioral observations for 226 individuals, and 863 parental food delivery observations for 112 nests, and reproductive success was known for 128 nests (Table 1).

### Among-individual and among-colony site repeatability

After controlling for other associated environmental covariates (see S1–S4 Tables), latency to enter a nest bearing a novel stimulus, the number of attacks towards a novel stimulus at the nest, and response to playbacks of conspecific alarm calls were significantly repeatable within individuals (random effect = Bird ID). The adjusted repeatability was low for Alarm call response (link scale *R* = 0.07, *P* = 0.04) but was higher for Number of attacks (link scale *R* = 0.12, *P* = 0.01) and higher still for Latency to enter nest (*R* = 0.20, *P* < 0.01).

**Fig 3:**
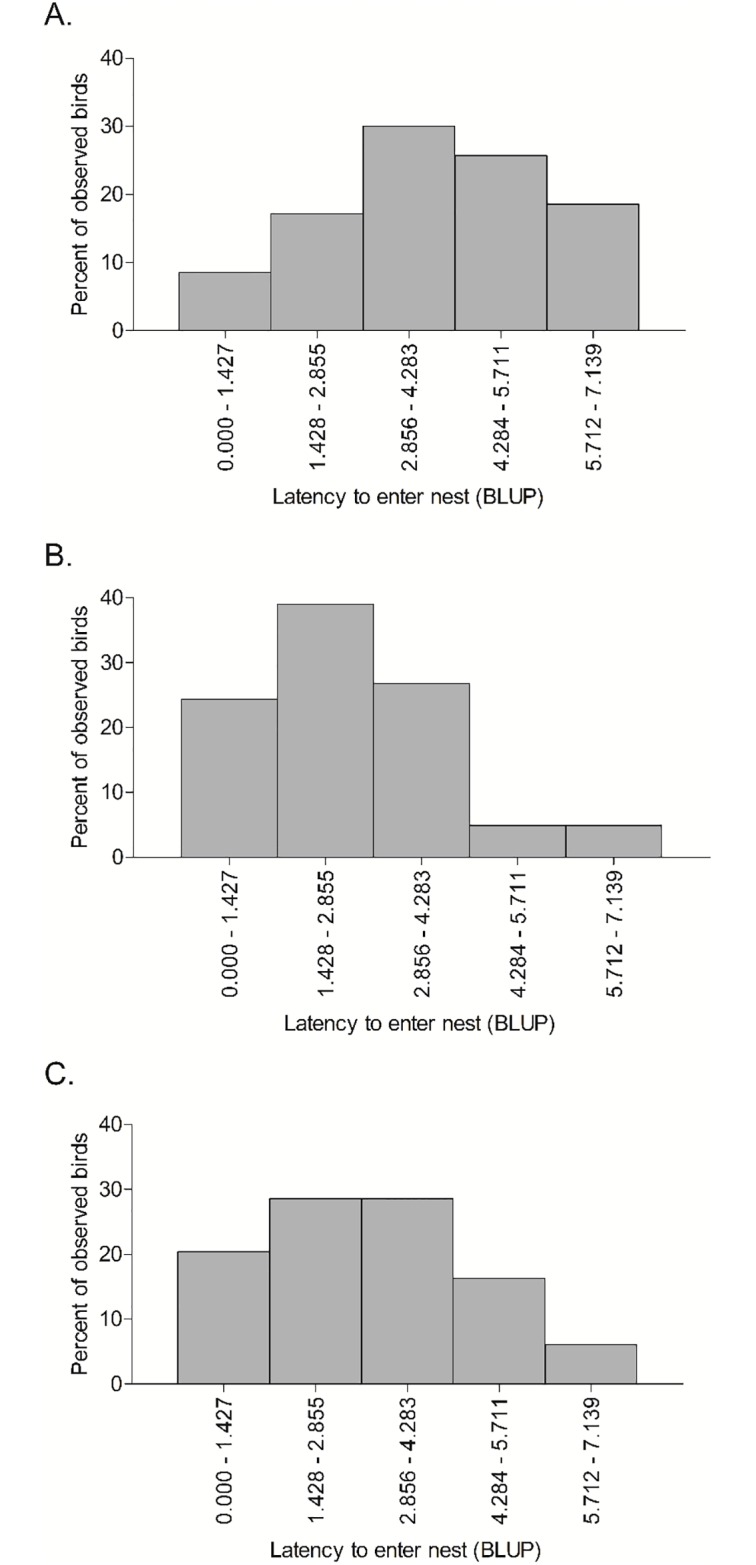
The distribution of individual relative scores (calculated as BLUPS) of latency to enter nest at three colony sites observed during 2017 and 2018 (years pooled at each site). Total number of birds at each colony site was 70 for (A) McDougals, 41 for (B) CR-1, and 49 for (C) Junkyard. Lower bin ranges represent shorter Latency to enter nest times.

Within-colony site (random = Site ID) repeatability was significant for Latency to enter nest (*R* = 0.13, *P* < 0.01); latency was longest for birds at McDougals (Table 1). Within-site repeatability was not significant for Number of attacks (link scale *R* = 0.01, *P* = 0.35), Alarm call response (link scale *R* < 0.01, *P* > 0.99), or the number of captures in a mist net (link scale *R* = 0.01, *P* = 0.42). The BLUPS of the Bird ID random effect for Latency to enter nest allowed us to visualize the personality composition of each colony site (Fig 3). The colony site that was associated with the longest individual Latency to enter nest scores was McDougals (BLUP $\bar{X}\pm \text{SE}=4.1\pm 0.21$) and the colony site with the shortest scores was CR-1 (BLUP $\bar{X}\pm \text{SE}=2.5\pm 0.24$), whereas scores at Junkyard fell in between (BLUP $\bar{X}\pm \text{SE}=3.0\pm 0.20$). Differences in the distribution of BLUP scores across all sites was significant (Chi-squared test: *X*^2^_8_ = 21.9, *P* = 0.01), but the distributions were not significantly different when comparing only CR-1 and Junkyard (Chi-squared test: $X_{4}^{2}=4.3$, *P* = 0.37).

### Behavioral correlations

We did not find a significant correlation between Latency to enter nest and Number of attacks (*r-cov*_LEN-NA_ = 0.11, HPD interval = -0.354–0.601) after controlling for other associated environmental covariates (see S5–S9 Tables). Similarly, we did not find a significant relationship between either behavioral measure and Number of captures (*r-cov*_LEN-NC_ = -0.06, HPD interval = -0.883 − 0.814; *r-cov*_NA-NC_ = 0.02, HPD interval = -0.513 − 0.548).

### Correlates of reproductive success and parental care

Reproductive success was not significantly correlated with Latency to enter nest (*r-cov*_LEN-RS_ = -0.01, HPD interval = -0.869 − 0.831) or Number of attacks (*r-cov*_NA-RS_ < 0.01, HPD interval = -0.572 − 0.493). In addition, nest-level scores of Latency to enter nest and Number of attacks were not significant predictors of parental care, measured as the number of food deliveries at a nest by both parents per hour (Table 2). However, nest-level mean Number of captures was a significant predictor of parental care such that nests with birds that were captured more also received more food deliveries (Table 2).

**Table 2:** Mixed model analysis of parental food deliveries, a nest-level measure of parental care in cliff swallows, in relation to potential life history, environmental, and behavioral predictor variables.

Variable	β (SE)	*P*
number of nestlings	21.5 (± 2.50)	< 0.01
nestling age	-71.1 (± 5.61)	< 0.01
nestling age^2^	-4.4 (± 0.35)	< 0.01
date	1.6 (± 0.43)	< 0.01
temperature (°C)	-0.4 (± 0.20)	0.04
wind speed (m/sec)	0.1 (± 0.18)	0.60
extent of sunshine (watts/m^2^)	0.3 (± 0.21)	0.11
Latency to enter nest	0.1 (± 0.30)	0.84
Number of attacks	-0.1 (± 0.21)	0.63
Number of captures	0.8 (± 0.34)	0.01

Nest ID and colony Site ID were modelled as random effects. *n*_nests_ = 112 and *n*_sites_ = 3.

## Discussion

Our study revealed significant among-individual and among-colony site variation in a cliff swallow’s latency to enter its nest when presented with a novel stimulus. We also found significant among-individual variation in the number of attacks directed toward a novel stimulus at the nest and in the response to broadcast of cliff swallow alarm call recordings, but among site variation in these measures was not significant. The behavioral measures were not correlated with one another or with the number of times an individual was captured by mist net. Differences among individuals in latency to enter the nest and the number of attacks were not significantly related to reproductive success or to the extent to which birds fed their nestlings. However, extent of nestling feeding was significantly predicted by the number of mist net captures, with pairs that were captured more on average also making more frequent food deliveries to the nest.

### Measures and correlates of personality

Despite evidence for relatively high repeatability in both behavioral measures that involved a reaction to a novel stimulus, we did not find support for a behavioral syndrome [5]. This suggests that Latency to enter nest and Number of attacks are independent facets of personality [55, 65, 66]. There is no consensus on which commonly identified personality axes are thought to be measured by behavioral tests involving novel objects or novel environments: some studies use novelty tests to measure personality along the avoidance-exploration axis [22, 67, 68], whereas others use novelty tests to measure personality along the shy-bold axis [65, 69–71]. In the case of novel item tests, the context in which the novel item is introduced may cause further inconsistencies in measured behaviors. For example, coyotes (*Canis latrans*) showed little avoidance toward a novel stimulus in unfamiliar surroundings but showed avoidance and neophobic reactions toward the same stimuli in familiar surroundings [35]. In our study, the novel stimulus was added to the focal bird’s own nest, a very familiar environment for the bird, and thus strong behavioral responses were expected. In this context, Latency to enter nest may be a measure of personality along the exploration-avoidance axis as the bird determines whether the novel stimulus is a threat, whereas Number of attacks may be a measure of personality along the shy-bold axis as the bird risks injury while responding to the novel stimulus. Number of attacks might alternatively reflect defensive aggression, which describes motor patterns exhibited by a socially aggressive animal but typically directed at a predator or threatening situation rather than a conspecific individual [72]. Regardless of which personality axes are represented, we can conclude that our measured behaviors are independent.

We were surprised that neither Latency to enter nest or Number of attacks were correlated with Number of captures, because the mist net, although perhaps less conspicuous, seemingly also acts as a novel stimulus or possibly a threat, at least after first capture (see [73]). After several successive days of netting at a colony site, cliff swallows learn to avoid mist nets, possibly because of the trauma associated with capture [73]. Active North American red squirrels (*Tamiasciurus hudsonicus*) were trapped significantly more frequently than less active squirrels [74]. Thus, Number of captures may be a measure of personality known as activity [22], which tends to generally describe an animal’s propensity to move.

Personality can affect both reproductive success and survival in some species [74–76, 60]. A meta-analysis found that exploration had a positive effect on survival and that boldness had a positive effect on reproductive success but a negative effect on survival [77]. The lack of an association in our study between Reproductive success and Latency to enter nest (possibly a measure of exploration) and Number of attacks (possibly a measure of boldness) may have been influenced by our sample size which was relatively small for a demographic study and may have reduced our ability to find a relationship between neophobia measures and reproductive success. For example, slight differences in fitness components (such as annual reproductive success), while evolutionarily significant over the long term, may often be indistinguishable empirically from null models due to a lack of power [78].

In some animals, more explorative individuals find food sources faster than less explorative individuals [79], and fast-exploration has been linked to increased nestling feeding rates and increased reproductive success [80]. However, Latency to enter nest was not a predictor of the number of food deliveries to a nest in cliff swallows. This lack of a relationship, as well as that for Number of attacks, may have resulted from pooling food deliveries by both parents to a nest and/or by using combined personality scores of both parents. This may have masked sex-differences in parental provisioning related to personality. For example, Mutzel et al. [80] found that fast-exploring female Eurasian blue tits (*Cyanistes caeruleus*) fed their offspring at higher rates, whereas exploratory personality of males was unrelated to nestling feeding rate. We found evidence in cliff swallows for a relationship between mean Number of captures and the number of parental food deliveries to a nest. Number of captures may be a measure of an individual’s activity personality such that individuals captured more often in mist nets are those that are most active near the nest. This may explain why these birds are also the ones that visit their nest more frequently with food if they are not traveling as far from the nest during foraging.

### Personality and coloniality

We did not find significant repeatability at the colony-site level for Number of attacks, Number of captures, or Alarm call response, suggesting that cliff swallows may not sort among colonies based on these behavioral measures. Both the rank order of neophobia trials and the rank order of alarm call trials were significant covariates, suggesting habituation to the novel stimulus and the alarm call play back [81]. Such habituation could reduce our ability to detect repeatable behavior at the colony-site level should habituation lead to reduced variability in the measured behavior across colonies.

The significant repeatability at the colony-site level for Latency to enter nest suggests that cliff swallows may sort into colonies based on this measure of personality. Individuals at the much larger CR-1 and Junkyard colonies were generally quicker to enter their nests when presented with a novel stimulus than individuals at the smaller McDougals colonies (Table 1, Fig 3). Our result contrasts with that of Dardenne et al. [12], who found higher levels of neophobia among barn swallows in larger colonies. They suggested that neophobic barn swallows may benefit from occupying a large colony where they can rely on other, more explorative individuals to lead them to food (c.f. [82]). If this scenario applies to cliff swallows, we would expect neophobic individuals to make fewer food deliveries, as they must wait to be led to food; however, we observed more frequent feeding visits at the small McDougals colonies compared to the larger colonies at CR-1 and Junkyard.

Increased predation odds at small versus large colonies may explain why more neophobic cliff swallows were found at the McDougals site. It is widely believed that predation risk of an individual is decreased when it occupies a large group [23–27, 83]. Without the safety in numbers afforded by large groups, animals in small groups may need to be more cautious to minimize predation risk, possibly explaining the increased neophobia in smaller cliff swallow colonies.

In great tits (*Parus major*), slow-exploring (neophobic) individuals were less aggressive toward conspecifics whereas fast-exploring individuals were more aggressive [84]. This relationship may also explain why cliff swallow individuals tended to be more neophobic at the smaller colony site. Not only were the CR-1 and Junkyard colonies much larger in size than at McDougals, but the nests at the larger colonies were also more densely packed (Fig 1), making avoidance of social interactions among neighboring individuals more difficult. A socially non-aggressive individual would be at a disadvantage in such a crowded colony where it would frequently need to fend off intruding neighbors [37]. Thus, there may be an advantage for neophobic individuals to choose small colonies where there is less opportunity for frequent social interaction.

Although cliff swallows might sort into different colony sites based on where they fall within the exploration-avoidance personality axis (as measured by Latency to enter nest), we cannot rule out that the observed behavioral variation among sites was instead shaped by the social environment after birds had already settled within a colony [85]. Behavioral plasticity shaped by changes in the social environment has been described in several birds [86–92], and most show a decrease in individual neophobia when in a group setting. King, Williams, and Mettke-Hofmann [93] found that individual Gouldian finches (*Erythrura gouldiae*) adjusted their boldness behavior to be more similar to that of their partner. We did not make comparisons of neophobia at the partner level over time, but on several occasions, a neophobia trial at the McDougals colony site elicited an almost colony-wide response, with several colony members from nearby nests hovering in front of the focal nest to inspect the novel stimulus. This collective response often occurred when the nest resident alarm-called in response to the piece of marking tape, and was not observed at the larger CR-1 or Junkyard colony sites. Bystanders at the McDougals site were possibly influenced by the alarm-calling (neophobic) nest resident, making bystanders more aware of the stimulus and potentially less likely to respond later when their own nests were tested. However, if this were the case, we should have seen overall shorter latencies to enter nest at the McDougals site compared to other sites.

In the only other study relating personality to colony size in cliff swallows, Roche and Brown [14] found some evidence for among-colony variation in vigilance behavior, but there was no clear relationship between vigilance level and colony size per se. While higher levels of neophobia in smaller colonies (this study) might lead to greater vigilance at those sites, vigilance can also reflect awareness of neighbors and the need to be alert to defend one’s nest from conspecifics, of which there are more in larger colonies. Possibly for this reason no systematic relationship between vigilance and colony size was detected [14].

We acknowledge some limitations to the present study. For example, the removal of ectoparasites, while necessary to increase the number of completed behavioral observations because of high nest failure rates due to swallow bug parasitism [37], might have altered the natural behavior of individuals in unknown ways. Perhaps the time necessary for parents to forage to provision offspring was reduced when nests were freed from parasitism [94, 95]. The laborious nature of these observations precluded conducting them at more colony sites, and thus we could not rigorously test the effect of colony size on individual behavior. However, we selected colony sites that were quite different in size while at the same time similar in other ways (e.g., all were in box-shaped concrete culverts; Fig 1), increasing the likelihood that observed differences among sites were related to colony size. Finally, given the highly social nature of cliff swallows, neophobia tests could not be conducted in isolation. As such, individuals may have seen the novel stimulus being presented at another nest nearby, and this may have happened more often than the protocol assumed. We know this occurred repeatedly at the McDougals site. Such unintended exposure (and resulting habituation) would have made us less likely to detect an effect of the novel stimulus, but we found the opposite result at McDougals, where neophobia was greater among residents.

### Conclusions

We were surprised to find only limited evidence in general of systematic variation in behavioral measures of neophobia and risk-taking among cliff swallows in different colonies. This may reflect the divergent and sometimes opposing selection pressures on behavior in different social environments. For example, bold (less neophobic) individuals could benefit in a larger colony by not fleeing at every alarm call and thus not frequently leaving their nest unattended and susceptible to theft of nesting material, egg loss, or brood parasitism from their many conspecific neighbors [37]. However, large colonies are also attacked by predators more often, to a degree that per capita predation risk is greatest in the very largest colonies [37]. Thus, bold individuals in a large colony, while minimizing interference from neighbors by not consistently reacting to alarm calls, might thus have a higher overall risk of predation. The result would be no net advantage for bold versus shy individuals in colonies of different sizes, and thus potentially no selection for bold or shy personalities in the first place. Future work should perhaps examine variation in other behavioral traits, such as foraging, in cliff swallow colonies of different sizes.

## Supporting information

S1 File Audio clips used. (PDF)

S2 File Univariate models for calculating repeatability. (PDF)

S3 File Bivariate models for calculating correlations. (PDF)

S4 File Parental food delivery data. (XLSX)

S5 File Behavioral data. (CSV)

S1 Table Univariate linear mixed model analysis of latency to enter a nest bearing a novel stimulus, a measure of neophobia in cliff swallows, in relation to potential life history and environmental predictor variables. (PDF)

S2 Table Univariate generalized linear mixed model analysis of the number of attacks towards a novel stimulus at the nest, a measure of neophobia in cliff swallows, in relation to potential life history and environmental predictor variables. (PDF)

S3 Table Univariate generalized linear mixed model analysis of the response to playbacks of conspecific alarm calls, a measure of risk-taking in cliff swallows, in relation to potential life history and environmental predictor variables. (PDF)

S4 Table Univariate generalized linear mixed model analysis of the number of captures, a measure of neophobia in cliff swallows, in relation to sex. (PDF)

S5 Table Bivariate mixed model analysis of latency to enter a nest bearing a novel stimulus and the number of attacks towards a novel stimulus at the nest, both measures of neophobia in cliff swallows, in relation to potential life history and environmental predictor variables. (PDF)

S6 Table Bivariate mixed model analysis of latency to enter a nest bearing a novel stimulus and the number of captures in a mist net placed at the colony, both measures of neophobia in cliff swallows, in relation to potential life history and environmental predictor variables. (PDF)

S7 Table Bivariate mixed model analysis of the number of attacks towards a novel stimulus at the nest and the number of captures in a mist net placed at the colony, both measures of neophobia in cliff swallows, in relation to potential life history and environmental predictor variables. (PDF)

S8 Table Bivariate mixed model analysis of latency to enter a nest bearing a novel stimulus, a measure of neophobia in cliff swallows, and reproductive success, measured as the number of nestlings surviving to 17 days of age, in relation to potential life history and environmental predictor variables. (PDF)

S9 Table Bivariate mixed model analysis of the number of attacks towards a novel stimulus at the nest, a measure of neophobia in cliff swallows, and reproductive success, measured as the number of nestlings surviving to 17 days of age, in relation to potential life history and environmental predictor variables. (PDF)

## References

[1] Bolnick D, Svanback R, Fordyce JA, Yang LH, Davis JM, Hulsey CD, et al The ecology of individuals: incidence and implications of individual specialization. Am Nat. 2003;161: 1–28. 10.1086/343878 12650459

[2] Farine DR, Montiglio PO, Spiegel O. From individuals to groups and back: the evolutionary implications of group phenotypic composition. Trends Ecol Evol. 2015;30: 609–621. 10.1016/j.tree.2015.07.005 26411618 PMC4594155

[3] Brown CR. The ecology and evolution of colony-size variation. Behav Ecol Sociobiol. 2016;70: 1613–1632.

[4] Dall SRX, Houston AI, McNamara JM. The behavioral ecology of personality: consistent individual differences from an adaptive perspective. Ecol Lett. 2004;7: 734–739.

[5] Sih A, Bell A, Johnson JC, Ziemba RE. Behavioral syndromes: an integrative overview. Q Rev Biol. 2004;79: 241–277. 10.1086/422893 15529965

[6] Pruitt JN, Reichart SE. How within-group behavioral variation and task efficiency enhance fitness in a social group. Proc R Soc Lond B Biol Sci. 2011;278: 1209–1215. 10.1098/rspb.2010.1700 PMC3049074 20943687

[7] Dall SR, Bell AM, Bolnick DI, Ratnieks FL. An evolutionary ecology of individual differences. Ecol Lett. 2012;15: 1189–1198. 10.1111/j.1461-0248.2012.01846.x 22897772 PMC3962499

[8] Pruitt JA, Grinsted L, Settepani V. Linking levels of personality: personalities of the ‘average’ and most ‘extreme’ group members predict colony-level personality. Anim Behav. 2013;86: 391–399.

[9] Bengston SE, Jandt JM. The development of collective personality: the ontogenetic drivers of behavioral variation across groups. Front Ecol Evol. 2014;10: 2:art81.

[10] Herbert-Read JE. Social behavior: the personalities of groups. Curr Biol. 2017;27: R1015–R1017. 10.1016/j.cub.2017.07.042 28950084

[11] Pruitt JN, Iturralde G, Avilés L, Riechart SE. Amazonian social spiders share similar within-colony behavioural variation and behavioural syndromes. Anim Behav. 2011;82: 1449–1455.

[12] Dardenne S, Ducatez S, Cote J, Poncin P, Stevens VM. Neophobia and social tolerance are related to breeding group size in a semi-colonial bird. Behav Ecol Sociobiol. 2013;67: 1317–1327.

[13] Liker A, Bokony V. Larger groups are more successful in innovative problem solving in house sparrows. Proc Natl Acad Sci U S A. 2009;106: 7893–7898. 10.1073/pnas.0900042106 19416834 PMC2683070

[14] Roche EA, Brown CR. Among-individual variation in vigilance at the nest in colonial cliff swallows. Wilson J Ornithol. 2013;125: 685–894.

[15] Brown CR., Brown MB. Heritable basis for choice of group size in a colonial bird. Proc Natl Acad Sci U S A. 2000;97: 14825–14830. 10.1073/pnas.97.26.14825 11121081 PMC19003

[16] Møller AP. Parent-offspring resemblance in degree of sociality in a passerine bird. Behav Ecol Sociobiol. 2002;51: 276–281.

[17] Keiser CN, Pruitt JN. Personality composition is more important than group size in determining collective foraging behavior in the wild. Proc R Soc Lond B Biol Sci. 2014;281: 20141424. 10.1098/rspb.2014.1424 PMC4213636 25320170

[18] Wright CM, Keiser CN, Pruitt JN. Colony personality composition alters colony-level plasticity and magnitude of defensive behavior in a social spider. Anim Behav. 2016;115: 175–183.

[19] Pinter-Wollman N, Mi B, Pruitt JN. Replacing bold individuals has a smaller impact on group performance than replacing shy individuals. Behav Ecol. 2017;28: 883–889.

[20] Brown CR, Stutchbury BJ, Walsh PD. Choice of colony size in birds. Trends Ecol Evol. 1990;5: 398–403. 10.1016/0169-5347(90)90023-7 21232400

[21] Wilson DS, Clark AB, Coleman K, Dearstyne T. Shyness and boldness in humans and other animals. Trends Ecol Evol. 1994;9: 442–446. 10.1016/0169-5347(94)90134-1 21236920

[22] Réale D, Reader SM, Sol D, McDougall PT, Dingemanse NJ. Integrating animal temperament within ecology and evolution. Biol Rev. 2007;82: 291–318. 10.1111/j.1469-185X.2007.00010.x 17437562

[23] Hamilton WD. Geometry for the selfish herd. J Theor Biol. 1971;31: 295–311. 10.1016/0022-5193(71)90189-5 5104951

[24] Alexander RD. The evolution of social behavior. Annu Rev Ecol Syst. 1974;5: 325–383.

[25] Pulliam HR, Millikan GC. Social organization in the non-reproductive season In: Farmer DS, King JR, Parkes KC, editors. Avian Biology. Vol. 6 New York: Academic Press; 1982 pp. 169–197.

[26] Elgar MA. Predator vigilance and group size in mammals and birds: a critical review of the empirical evidence. Biol Rev. 1989;64: 13–33. 10.1111/j.1469-185x.1989.tb00636.x 2655726

[27] Brown CR, Brown MB. Avian coloniality: progress and problems. Curr Ornithol. 2001;16: 1–82.

[28] Treisman M. Predation and the evolution of gregariousness. II. An economic model for predator-prey interactions. Anim Behav. 1975;23: 801–825.

[29] Beauchamp G. Determinants of false alarms in staging flocks of semipalmated sandpipers. Behav Ecol. 2010;21: 584–587.

[30] Beauchamp G, Ruxton GD. False alarms and the evolution of antipredator vigilance. Anim Behav. 2007;75: 1199–1206.

[31] Pollard KA. Making the most of alarm signals: the adaptive value of individual discrimination in an alarm context. Behav Ecol. 2011;22: 93–100.

[32] DuRant SE, Hopkins WA, Hepp GR, Walters JR. Ecological, evolutionary, and conservation implications of incubation temperature-dependent phenotypes in birds. Biol Rev. 2013;88: 499–509. 10.1111/brv.12015 23368773

[33] Sheppe W. Exploration by the deermouse, *Peromyscus leucopus*. Am Midl Nat. 1966;76: 257–276.

[34] Greenburg R, Mettke-Hofmann C. Ecological aspects of neophobia and neophilia in birds. Curr Ornithol. 2001;16: 119–178.50 Bates D, Maechler M, Bolker B, Walker S. Fitting linear mixed-effects models using lme4. J Stat Softw. 2015;67: 1–48.

[35] Harris CE, Knowlton FF. Differential responses of coyotes to novel stimuli in familiar and unfamiliar settings. Can J Zool. 2001;79: 2005–2013.

[36] Brown CR, Brown MB, Roche EA. Spatial and temporal unpredictability of colony size in cliff swallows across 30 years. Ecol Monogr. 2013;83: 511–530.

[37] Brown CR, Brown MB. Coloniality in the cliff swallow: the effect of group size on social behavior. Chicago: University of Chicago Press; 1996.

[38] Brown CR, Brown MB. Testis size increases with colony size in cliff swallows. Behav Ecol. 2003;14: 569–575.

[39] Brown CR, Brown MB, Raouf SA, Smith LC, Wingfield JC. Steroid hormone levels are related to choice of colony size in cliff swallows. Ecol. 2005;86: 2904–2915.

[40] Smith LC, Raouf SA, Brown MB, Wingfield JC, Brown CR. Testosterone and group size in cliff swallows: testing the “challenge hypothesis” in a colonial bird. Horm Behav. 2005;47: 76–82. 10.1016/j.yhbeh.2004.08.012 15579268

[41] Brown CR, Roche EA, Brown MB. Variation in age composition among colony sizes in cliff swallows. J Field Ornithol. 2014;85: 289–300. 10.1111/jofo.12068 29628606 PMC5884171

[42] Ringsby TH, Berge T, Saether BE, Jensen H. Reproductive success and individual variation in feeding frequency of House Sparrows (*Passer domesticus*). J Ornithol. 2009;150: 469–481.

[43] Brown CR, Brown MB. Ectoparasitism as a cost of coloniality in cliff swallows (*Hirundo pyrrhonota*). Ecol. 1986;67: 1206–1218.

[44] Brown CR, Brown MB. Empirical measurement of parasite transmission between groups in a colonial bird. Ecol. 2004;85: 1619–1626.

[45] Runjaic J, Bellovich IJ, Brown CR, Booth W. No detectable insecticide resistance in swallow bugs (Hemiptera: Cimicidae) following long-term exposure to naled (Dibrom 8). J Med Entomol. 2017;54: 994–998. 10.1093/jme/tjw230 28399289

[46] Brown CR, Brown MB, Pyle P, Patten MA. Cliff swallow (*Petrochelidon pyrrhonota*) In Rodewald PG, editor. The birds of North America. Ithaca: Cornell Lab of Ornithology; 2017.

[47] Betini GS, Norris DR. The relationship between personality and plasticity in tree swallow aggression and the consequences for reproductive success. Anim Behav. 2012;83: 137–143.

[48] Dingemanse NJ, Dochtermann NA. Quantifying individual variation in behavior: mixed-effect modeling approaches. J Anim Ecol. 2013;82: 39–54. 10.1111/1365-2656.12013 23171297

[49] Warton DI, Hui FKC. The arcsine is asinine: the analysis of proportions in ecology. Ecol. 2011;92: 3–10. 10.1890/10-0340.1 21560670

[50] Bates D, Maechler M, Bolker B, Walker S. Fitting linear mixed-effects models using lme4. J Stat Softw. 2015;67: 1–48.

[51] Sih A, Bell A, Johnson JC. Behavioral syndromes: an ecological and evolutionary overview. Trends Ecol Evol. 2004;19: 372–378. 10.1016/j.tree.2004.04.009 16701288

[52] Bell AM, Hankison SJ, Laskowski KL. The repeatability of behavior: a meta-analysis. Anim Behav. 2009;77: 771–783. 10.1016/j.anbehav.2008.12.022 24707058 PMC3972767

[53] Nakagawa S, Schielzeth H. Repeatability for Gaussian and non-Gaussian data: a practical guide for biologists. Biol Rev. 2010;85: 935–956. 10.1111/j.1469-185X.2010.00141.x 20569253

[54] Stoffel MA, Nakagawa S, Schielzeth H. rptR: repeatability estimation and variance decomposition by generalized linear mixed-effects models. Methods Ecol Evol. 2017;8: 1639–1644.

[55] Carter AJ, Feeney WE, Marshall HH, Cowlishaw G, Heinsohn R. Animal personality: what are behavioral ecologists measuring? Biol Rev. 2013;88: 465–475. 10.1111/brv.12007 23253069

[56] Hadfield JD. MCMC methods for multi-response generalized mixed models: the MCMCglmm R package. J Stat Softw. 2010;33: 1–25.20808728

[57] Hadfield JD, Wilson AJ, Garant D, Sheldon BC, Kruuk LE. The misuse of BLUP in ecology and evolution. Am Nat. 2010;175: 116–125. 10.1086/648604 19922262

[58] Houslay TM, Wilson AJ. Avoiding the misuse of BLUP in behavioral ecology. Behav Ecol. 2017;28: 948–952. 10.1093/beheco/arx023 29622923 PMC5873244

[59] Pinheiro JC, Bates DM. Mixed-effects models in S and S-PLUS. New York: Springer; 2000.

[60] Boon AK, Réale D, Boutin S. The interaction between personality, offspring fitness, and food abundance in North American red squirrels. Ecol Lett. 2007;10: 1094–1104. 10.1111/j.1461-0248.2007.01106.x 17877738

[61] Martin JG, Réale D. Temperament, risk assessment and habituation to novelty in eastern chipmunks, *Tamius striatus*. Anim Behav. 2008;75: 309–318.

[62] SAS Institute. SAS/STAT user’s guide, version 9.1 Cary: SAS Institute; 2004.

[63] R Core Team. R: a language and environment for statistical computing. Vienna: R Foundation for Statistical Computing; 2018.

[64] Grueber CE, Nakagawa S, Laws RJ, Jamieson IG. Multimodel inference in ecology and evolution: challenges and solutions. J Evol Biol. 2011;24: 699–711. 10.1111/j.1420-9101.2010.02210.x 21272107

[65] Burns JG. The validity of three tests of temperament in guppies (*Poecilia reticulate*). J Comp Psychol. 2008;122: 344–356. 10.1037/0735-7036.122.4.344 19014258

[66] Carter AJ, Marshall HH, Heinsohn R, Cowlishaw G. How not to measure boldness: novel object and antipredator responses are not the same in wild baboons. Anim Behav. 2012;84: 603–609.

[67] Stӧwe M, Kotrschal K. Behavioural phenotypes may determine whether social context facilitates or delays novel object exploration in ravens (*Corvus corax*). J Ornithol. 2007;148: 179–184.

[68] Kurvers RHJM, Eijkelenkamp B, van Oers K, van Lith B, van Wieren SE, Ydenberg RC, et al Personality differences explain leadership in barnacle geese. Anim Behav. 2009;78: 447–453.

[69] Coleman K, Wilson DS. Shyness and boldness in pumpkinseed sunfish: individual differences are context-specific. Anim Behav. 1998;56: 927–936. 10.1006/anbe.1998.0852 9790704

[70] Toms CN, Echevarria DJ, Jouandot DJ. How not to measure boldness: novel object and antipredator responses are not the same in wild baboons. Anim Behav. 2012;84: 603–609.

[71] Blaszczyk MB. Boldness towards novel objects predicts predator inspection in wild vervet monkeys. Anim Behav. 2017;123: 91–100.

[72] Blumstein DT, Petelle MB, Wey TW. Defensive and social aggression: repeatable but independent. Behav Ecol. 2013;24: 457–461.

[73] Roche EA, Brown CR, Brown MB, Lear KM. Recapture heterogeneity in cliff swallows: increased exposure to mist nets leads to net avoidance. PLoS One. 2013;8: e58092 10.1371/journal.pone.0058092 23472138 PMC3589455

[74] Boon AK, Réale D, Boutin S. Personality, habitat use, and their consequences for survival in North American red squirrels *Tamiasciurus hudsonicus*. Oikos. 2008;117: 1321–1328.

[75] Dingemanse NJ, Both C, Drent PJ, Tinbergen JM. Fitness consequences of avian personalities in a fluctuating environment. Proc R Soc Lond B Biol Sci. 2004;271: 847–852. 10.1098/rspb.2004.2680 PMC1691663 15255104

[76] Patrick SC, Weimerskirch H. Personality, foraging and fitness consequences in a long lived seabird. PLoS One. 2014;4: e87269. 10.1371/journal.pone.0087269 PMC3913606 24504180

[77] Smith BR, Blumstein DT. Fitness consequences of personality: a meta-analysis. Behav Ecol. 2008;19: 448–455.

[78] Steiner UK, Tuljapurkar S. Neutral theory for life histories and individual variability in fitness components. Proc Natl Acad Sci U S A. 2012;109: 4684–4689. 10.1073/pnas.1018096109 22392997 PMC3311353

[79] Jolles JW, Boogert NJ, Sridhar VH, Couzin ID, Manica A. Consistent individual differences drive collective behavior and group functioning of schooling fish. Curr Biol. 2017;27: 2862–2868. 10.1016/j.cub.2017.08.004 28889975 PMC5628957

[80] Mutzel A, Dingemanse NJ, Araya-Ajoy YG, Kempenaers B. Parental provisioning behavior plays a key role in linking personality with reproductive success. Proc R Soc Lond B Biol Sci. 2013;280: 20131019. 10.1098/rspb.2013.1019 PMC3712423 23782885

[81] Groves PM, Thompson RF. Habituation: a dual-process theory. Psychol Rev. 1970;5: 419–450. 10.1037/h0029810 4319167

[82] Hebblethwaite ML, Shields WM. Social influences on barn swallow foraging in the Adirondacks: a test of competing hypotheses. Anim Behav. 1990;39: 97–104.

[83] Brown CR, Hoogland JL. Risk in mobbing for solitary and colonial swallows. Anim Behav. 1986;34: 1319–1323.

[84] Verbeek EM, Boon A, Drent PJ. Exploration, aggressive behavior and dominance in pair-wise confrontations of juvenile male great tits. Behav. 1996;133: 945–963.

[85] Webster MM, Ward AJW. Personality and social context. Biol Rev. 2011;86: 759–773. 10.1111/j.1469-185X.2010.00169.x 21091603

[86] Coleman SL, Mellgren RL. Neophobia when feeding alone or in flocks in zebra finches, *Taeniopygia guttata*. Anim Behav. 1994;48: 903–907.

[87] van Oers K, Klunder M, Drent PJ. Context dependence of personalities: risk-taking behavior in a social and a nonsocial situation. Behav Ecol. 2005;16: 716–723.

[88] Stӧwe M, Bugnyar T, Heinrich B, Kotrschal K. Effects of group size on approach to novel objects in ravens (*Corvus corax*). Ethology. 2006;112: 1079–1088.

[89] Schuett W, Dall SRX. Sex differences, social context and personality in zebra finches, *Taeniopygia guttata*. Anim Behav. 2009;77: 1041–1050.

[90] Mainwaring MC, Beal JL, Hartley IR. Zebra finches are bolder in an asocial rather than social context. Behav Processes. 2011;87: 171–175. 10.1016/j.beproc.2011.03.005 21443934

[91] Griffin AS, Lermite F, Perea M, Guez D. To innovate or not: contrasting effects of social groupings on safe and risky foraging in Indian mynahs. Anim Behav. 2013;86: 1291–1300.

[92] Kerman K, Miller L, Sewall K. The effect of social context on measures of boldness: zebra finches (*Taeniopygia guttata*) are bolder when housed individually. Behav Processes. 2018;157: 18–23. 10.1016/j.beproc.2018.08.007 30145276

[93] King AJ, Williams LJ, Mettke-Hofmann C. The effects of social conformity on Gouldian finch personality. Anim Behav. 2015;99: 25–31.

[94] Tripet F, Richner H. Host responses to ectoparasites: food compensation by parent blue tits. Oikos. 1997;78: 557–561.

[95] Hurtrez-Boussès S, Blondel J, Perret P, Fabreguettes J, Renaud F. Chick parasitism by blowflies affects feeding rates in a Mediterranean population of blue tits. Ecol Lett. 1998;1: 17–20.

