# Supplementary material for: Variation in neophobia among cliff swallows at different colonies
Source: PLoS One. 2019 Dec 23;14(12):e0226886. doi: 10.1371/journal.pone.0226886 (PMC6927619; doi:10.1371/journal.pone.0226886)
Supplement: S1 File — (PDF) [file pone.0226886.s001.pdf]

S1 File: Audio clips used.

2017 Alarm Calls:

- A = from Macaulay Library at the Cornell Lab of Ornithology: ML Audio 66819; clip span 0:25 – 0:40
- B = from Macaulay Library at the Cornell Lab of Ornithology: ML Audio 210542; clip span 0:00 – 0:15
- C = from the Audubon Bird Guide application: Cliff swallow alarm call

2018 Alarm Calls:

All from Macaulay Library at the Cornell Lab of Ornithology

- D = ML Audio 111053; clip span 0:05 – 0:20
- E = ML Audio 210542; clip span 0:20 – 0:35
- F = ML Audio 44543; clip span 0:25 – 0:40

Link to Macaulay Library at the Cornell Lab of Ornithology: https://www.macaulaylibrary.org/
