# Supplementary material for: Variation in neophobia among cliff swallows at different colonies
Source: PLoS One. 2019 Dec 23;14(12):e0226886. doi: 10.1371/journal.pone.0226886 (PMC6927619; doi:10.1371/journal.pone.0226886)
Supplement: S2 File — (PDF) [file pone.0226886.s002.pdf]

S2 File: Univariate models for calculating repeatability.

Univariate Model 1: Latency to enter nest (LEN)

UModel1 <- lmer(logitLEN ~ SEX + TRIALRANK + TEMP + WINDSP + SUNSHINE + DAYSSINCELAID + (1|BIRDID) + (1|SITEID), data = Neophobia)

LEN_Repeatability <- rpt(logitLEN ~ SEX + TRIALRANK + TEMP + WINDSP + SUNSHINE + DAYSSINCELAID + (1|BIRDID) + (1|SITEID), grname = c("BIRDID", "SITEID", "Fixed"), data = Neophobia, datatype = "Gaussian", nboot = 1000, npermut = 0)

Univariate Model 2: Number of attacks (NA)

UModel2 <- glmer(NA ~ SEX + TRIALRANK + LEN + TEMP + WINDSP + SUNSHINE + DAYSSINCELAID + (1|BIRDID) + (1|SITEID), family = poisson, data = Neophobia)

NA_Repeatability <- rpt(NA ~ SEX + TRIALRANK + LEN + TEMP + WINDSP + SUNSHINE + DAYSSINCELAID + (1|BIRDID) + (1|SITEID), grname = c("BIRDID", "SITEID", "Fixed"), data = Neophobia, datatype = "Poisson", nboot = 1000, npermut = 0)

Univariate Model 3: Alarm call response (ACR)

UModel3 <- glmer(ACR ~ SEX + TRIALRANK + STATUSBEFORE + TEMP + WINDSP + SUNSHINE + DAYSSINCELAID + (1|BIRDID) + (1|SITEID), family = binomial, data = Alarm)

ACR_Repeatability <- rpt(ACR ~ SEX + TRIALRANK + STATUSBEFORE + TEMP + WINDSP + SUNSHINE + DAYSSINCELAID + (1|BIRDID) + (1|SITEID), grname = c("BIRDID", "SITEID", "Fixed"), data = Alarm, datatype = "Binary", nboot = 1000, npermut = 0)

Univariate Model 4: Number of captures (NC)

UModel4 <- glmer(NC ~ SEX + (1|SITEID), family = poisson, data = NumCaptures)

NC_Repeatability <- rpt(NC ~ SEX + (1|SITEID), grname = c("SITEID", "Fixed"), data = NumCaptures, datatype = "Poisson", nboot = 1000, npermut = 0)
