# Supplementary material for: Variation in neophobia among cliff swallows at different colonies
Source: PLoS One. 2019 Dec 23;14(12):e0226886. doi: 10.1371/journal.pone.0226886 (PMC6927619; doi:10.1371/journal.pone.0226886)
Supplement: S3 File — (PDF) [file pone.0226886.s003.pdf]

S3 File: Bivariate models for calculating correlations.

Bivariate Model 1: Are Latency to enter nest (LEN) and Number of attacks (NA) correlated?

```
prior1 = list(G=list(G1=list(V=diag(c(1,1),2),nu=1.002),
                     G2=list(V=diag(c(1,1),2),nu=1.002)),
                     R=list(V=diag(2),nu=1.002))

BModel1 <- MCMCglmm(cbind(logitLEN, NA) ~
                    at.level(trait,1):(SEX) + at.level(trait,1):(TRIALRANK) +
                    at.level(trait,1):(TEMP) + at.level(trait,1):(WINDSP) +
                    at.level(trait,1):(SUNSHINE) + at.level(trait,1):(DAYSSINCELAID) +
                    at.level(trait,2):(SEX) + at.level(trait,2):(TRIALRANK) +
                    at.level(trait,2):(TEMP) + at.level(trait,2):(WINDSP) +
                    at.level(trait,2):(SUNSHINE) + at.level(trait,2):( DAYSSINCELAID) +
                    at.level(trait,2):(LEN) - 1,
                    random =~ us(trait):BIRDID + us(trait):SITEID,
                    rcov =~ us(trait):units,
                    family = c("gaussian","poisson"),
                    prior = prior1,
                    nitt=750000, burn=50000, thin=175,
                    verbose = TRUE,
                    data = Neophobia))
```

Bivariate Model 2: Are Latency to enter nest (LEN) and Number of captures (NC) correlated?

```
prior2 = list(G=list(G1=list(V=diag(c(1,0.0001),2),nu=1.002, fix = 2),
                     G2=list(V=diag(c(1,0.0001),2),nu=1.002, fix = 2)),
                     R=list(V=diag(2),nu=1.002))

BModel2 <- MCMCglmm(cbind(logitLEN, NC) ~
                    at.level(trait,1):(SEX) + at.level(trait,1):(TRIALRANK) +
                    at.level(trait,1):(TEMP) + at.level(trait,1):(WINDSP) +
                    at.level(trait,1):(SUNSHINE) + at.level(trait,1):(DAYSSINCELAID) +
                    at.level(trait,2):(SEX) - 1,
                    random =~ us(trait):BIRDID + us(trait):SITEID,
                    rcov =~ us(trait):units,
                    family = c("gaussian","poisson"),
                    prior = prior2,
                    nitt=750000, burn=50000, thin=175,
                    verbose = TRUE,
                    data = Neophobia))
```

Bivariate Model 3: Are Number of attacks (NA) and Number of captures (NC) correlated?

```
prior3 = list(G=list(G1=list(V=diag(c(1,0.0001),2),nu=1.002, fix = 2),
                     G2=list(V=diag(c(1,0.0001),2),nu=1.002, fix = 2)),
                     R=list(V=diag(2),nu=1.002))

BModel3 <- MCMCglmm(cbind(NUMCHARGES1, NUMCAPTURES) ~
                     at.level(trait,1):(SEX) + at.level(trait,1):(TRIALRANK) +
                     at.level(trait,1):(TEMP) + at.level(trait,1):(WINDSP) +
                     at.level(trait,1):(SUNSHINE) + at.level(trait,1):(DAYSSINCELAID) +
                     at.level(trait,1):(LEN) + at.level(trait,2):(SEX) - 1,
                     random =~ us(trait):BIRDID + us(trait):SITEID,
                     rcov =~ us(trait):units,
                     family = c("poisson","poisson"),
                     prior = prior3,
                     nitt=750000, burn=50000, thin=175,
                     verbose = TRUE,
                     data = Neophobia))
```

Bivariate Model 4: Are Latency to enter nest (LEN) and Reproductive success (RS) correlated?

```
prior4= list(G=list(G1=list(V=diag(c(1,0.0001),2),nu=1.002, fix = 2),
                     G2=list(V=diag(c(1,0.0001),2),nu=1.002, fix = 2)),
                     R=list(V=diag(2),nu=1.002))

BModel4 <- MCMCglmm(cbind(logitLEN, RS) ~
                     at.level(trait,1):(SEX) +  at.level(trait,1):(TRIALRANK) +
                     at.level(trait,1):(TEMP) + at.level(trait,1):(WINDSP) +
                     at.level(trait,1):(SUNSHINE) + at.level(trait,1):(DAYSSINCELAID) +
                     at.level(trait,2):(LAYDATE) + at.level(trait,2):(CLUTCHSIZE) - 1,
                     random =~ us(trait):BIRDID + us(trait):SITEID,
                     rcov =~ us(trait):units,
                     family = c("gaussian","gaussian"),
                     prior = prior4,
                     nitt=750000, burn=50000, thin=175,
                     verbose = TRUE,
                     data = Neophobia))
```

Bivariate Model 5: Are Number of attacks (NA) and Reproductive success (RS) correlated?

```
prior5 = list(G=list(G1=list(V=diag(c(1,0.0001),2),nu=1.002, fix = 2),
                     G2=list(V=diag(c(1,0.0001),2),nu=1.002, fix = 2)),
                     R=list(V=diag(2),nu=1.002))

BModel5 <- MCMCglmm(cbind(NA, RS) ~
                at.level(trait,1):(SEX) + at.level(trait,1):(TRIALRANK) +
                at.level(trait,1):(TEMP) + at.level(trait,1):(WINDSP) +
                at.level(trait,1):(SUNSHINE) + at.level(trait,1):(DAYSSINCELAID) +
                at.level(trait,1):(LEN) + at.level(trait,2):(LAYDATE) +
                at.level(trait,2):(CLUTCHSIZE) - 1,
                random =~ us(trait):BIRDID + us(trait):SITEID,
                rcov =~ us(trait):units,
                family = c("poisson","gaussian"),
                prior = prior5,
                nitt=750000, burnin =50000, thin=175,
                verbose = TRUE,
                data = Neophobia))
```
