# Supplementary material for: Variation in neophobia among cliff swallows at different colonies
Source: PLoS One. 2019 Dec 23;14(12):e0226886. doi: 10.1371/journal.pone.0226886 (PMC6927619; doi:10.1371/journal.pone.0226886)
Supplement: S1 Table — (PDF) [file pone.0226886.s006.pdf]

S1 Table: Univariate linear mixed model analysis of latency to enter a nest bearing a novel stimulus, a measure of neophobia in cliff swallows, in relation to potential life history and environmental predictor variables.

Covariate	Estimate	SE	t-value
Intercept	2.2617	1.0288	2.198
Sex[a]	-0.3029	0.4969	-0.610
Trial rank order 2[b]	-2.2195	0.4290	-5.174
Trial rank order 3[b]	-2.8804	0.4872	-5.912
Trial rank order 4[b]	-2.6596	0.6463	-4.115
Temperature (°C)	-0.2109	0.1811	-1.165
Wind speed (m/sec)	0.0379	0.1752	0.216
Extent of sunshine (watts/m^2)	0.1480	0.1782	0.831
Days since 1st egg laid	0.3063	0.2573	1.190

Number of observations: 533; Bird ID and colony Site ID were modelled as a random effects. $n_{ind.}$ = 160 and n_{sites} = 3.

[a] In relation to female as baseline.

[b] In relation to trial rank order 1 as baseline.
