# Supplementary material for: Variation in neophobia among cliff swallows at different colonies
Source: PLoS One. 2019 Dec 23;14(12):e0226886. doi: 10.1371/journal.pone.0226886 (PMC6927619; doi:10.1371/journal.pone.0226886)
Supplement: S2 Table — (PDF) [file pone.0226886.s007.pdf]

S2 Table: Univariate generalized linear mixed model analysis of the number of attacks towards a novel stimulus at the nest, a measure of neophobia in cliff swallows, in relation to potential life history and environmental predictor variables.

Covariate	Estimate	SE	Z-value	p-value
Intercept	0.975	0.103	9.464	< 0.0001
Sex[a]	-0.078	0.089	-0.881	0.3782
Trial rank order 2[b]	-0.045	0.071	-0.630	0.5284
Trial rank order 3[b]	-0.113	0.086	-1.305	0.1919
Trial rank order 4[b]	-0.198	0.116	-1.696	0.0898
Latency to enter nest	0.182	0.033	5.479	< 0.0001
Temperature (°C)	-0.089	0.030	-2.938	0.0033
Wind speed (m/sec)	0.046	0.030	1.541	0.1234
Extent of sunshine (watts/m^2)	-0.012	0.030	-0.398	0.6905
Days since 1st egg laid	-0.006	0.046	-0.127	0.8990

Number of observations: 533; Bird ID and colony Site ID were modelled as a random effects. $n_{ind.}$ = 160 and n_{sites} = 3.

[a] In relation to female as baseline.

[b] In relation to trial rank order 1 as baseline.
