# Supplementary material for: Variation in neophobia among cliff swallows at different colonies
Source: PLoS One. 2019 Dec 23;14(12):e0226886. doi: 10.1371/journal.pone.0226886 (PMC6927619; doi:10.1371/journal.pone.0226886)
Supplement: S3 Table — (PDF) [file pone.0226886.s008.pdf]

S3 Table: Univariate generalized linear mixed model analysis of the response to playbacks of conspecific alarm calls, a measure of risk-taking in cliff swallows, in relation to potential life history and environmental predictor variables.

Covariate	Estimate	SE	Z-value	p-value
Intercept	-2.646	0.258	-10.236	< 0.0001
Sex[a]	0.269	0.259	1.040	0.2983
Trial rank order	-0.836	0.141	-5.932	< 0.0001
Status before alarm[b]	1.560	0.236	6.602	< 0.0001
Temperature (°C)	-0.068	0.107	-0.633	0.5266
Wind speed (m/sec)	-0.135	0.111	-1.211	0.2261
Extent of sunshine (watts/m^2)	-0.250	0.095	-2.629	0.0086
Days since 1st egg laid	-0.305	0.128	-2.381	0.0173

Number of behavioral observations: 851; Bird ID and colony Site ID were modelled as a random effects. $n_{ind.}$ = 225 and n_{sites} = 3.

[a] In relation to female as baseline.

[b] In relation to status before = in nest but not visible to observer as baseline.
