# Supplementary material for: Variation in neophobia among cliff swallows at different colonies
Source: PLoS One. 2019 Dec 23;14(12):e0226886. doi: 10.1371/journal.pone.0226886 (PMC6927619; doi:10.1371/journal.pone.0226886)
Supplement: S4 Table — (PDF) [file pone.0226886.s009.pdf]

S4 Table: Univariate generalized linear mixed model analysis of the number of captures, a measure of neophobia in cliff swallows, in relation to sex.

Covariate	Estimate	SE	Z-value	p-value
Intercept	0.249	0.132	1.882	0.0598
Sex[a]	-0.035	0.156	-0.222	0.8242

Number of behavioral observations: 160; colony Site ID was modelled as a random effect, n_{sites} = 3.

[a] In relation to female as baseline.
