# Supplementary material for: Variation in neophobia among cliff swallows at different colonies
Source: PLoS One. 2019 Dec 23;14(12):e0226886. doi: 10.1371/journal.pone.0226886 (PMC6927619; doi:10.1371/journal.pone.0226886)
Supplement: S5 Table — (PDF) [file pone.0226886.s010.pdf]

S5 Table: Bivariate mixed model analysis of latency to enter a nest bearing a novel stimulus and the number of attacks towards a novel stimulus at the nest, both measures of neophobia in cliff swallows, in relation to potential life history and environmental predictor variables.

Behavioral measure	Covariate	Post.mean	L CI	U CI	Eff. samp	pMCMC
Latency to enter nest	Female	2.312	-2.300	6.466	4660	0.161
	Male	1.940	-2.749	5.936	4716	0.192
	Trial rank order 2[a]	-2.231	-3.136	-1.381	4000	< 0.0001
	Trial rank order 3[a]	-2.825	-3.767	-1.810	4000	< 0.0001
	Trial rank order 4[a]	-2.392	-3.924	-0.922	4000	0.003
	Temperature (°C)	-0.167	-0.542	0.206	3627	0.376
	Wind speed (m/sec)	0.042	-0.320	0.391	3766	0.827
	Extent of sunshine (watts/m^2)	0.188	-0.160	0.560	4000	0.318
	Days since 1st egg laid	0.258	-0.272	0.760	4000	0.343
Number of attacks	Female	0.935	-0.999	2.774	4000	0.174
	Male	0.879	-0.923	2.855	4000	0.181
	Trial rank order 2[a]	-0.115	-0.348	0.118	4000	0.334
	Trial rank order 3[a]	-0.176	-0.454	0.121	4000	0.210
	Trial rank order 4[a]	-0.347	-0.692	0.015	4000	0.051
	Temperature (°C)	-0.093	-0.175	-0.018	4175	0.021
	Wind speed (m/sec)	0.046	-0.032	0.119	4000	0.243
	Extent of sunshine (watts/m^2)	-0.007	-0.084	0.071	4000	0.856
	Days since 1st egg laid	0.013	-0.091	0.114	4000	0.809
	Latency to enter nest	0.067	-0.244	0.376	3776	0.666

Number of observations: 533; Bird ID and colony Site ID were modelled as a random effects.
$n_{ind.}$ = 160 and n_{sites} = 3.
[a] In relation to trial rank order 1 as baseline.
