# Supplementary material for: Variation in neophobia among cliff swallows at different colonies
Source: PLoS One. 2019 Dec 23;14(12):e0226886. doi: 10.1371/journal.pone.0226886 (PMC6927619; doi:10.1371/journal.pone.0226886)
Supplement: S6 Table — (PDF) [file pone.0226886.s011.pdf]

S6 Table: Bivariate mixed model analysis of latency to enter a nest bearing a novel stimulus and the number of captures in a mist net placed at the colony, both measures of neophobia in cliff swallows, in relation to potential life history and environmental predictor variables.

Behavioral measure	Covariate	Post.mean	L CI	U CI	Eff. samp	pMCMC
Latency to enter nest	Female	2.223	-0.720	5.039	4000	0.101
	Male	1.885	-0.967	4.782	4000	0.132
	Trial rank order 2[a]	-2.226	-3.041	-1.368	4000	< 0.0001
	Trial rank order 3[a]	-2.827	-3.814	-1.794	4000	< 0.0001
	Trial rank order 4[a]	-2.353	-3.899	-0.932	4075	0.002
	Temperature (°C)	-0.173	-0.564	0.189	4000	0.362
	Wind speed (m/sec)	0.043	-0.308	0.399	4000	0.817
	Extent of sunshine (watts/m^2)	0.187	-0.164	0.563	4000	0.316
	Days since 1st egg laid	0.247	-0.310	0.737	4091	0.361
Number of captures	Female	0.260	0.123	0.383	3822	< 0.0001
	Male	0.203	0.095	0.301	3664	< 0.0001

Number of observations: 533; Bird ID and colony Site ID were modelled as a random effects.
$n_{ind.}$ = 160 and n_{sites} = 3.
[a] In relation to trial rank order 1 as baseline.
