# Supplementary material for: Variation in neophobia among cliff swallows at different colonies
Source: PLoS One. 2019 Dec 23;14(12):e0226886. doi: 10.1371/journal.pone.0226886 (PMC6927619; doi:10.1371/journal.pone.0226886)
Supplement: S7 Table — (PDF) [file pone.0226886.s012.pdf]

S7 Table: Bivariate mixed model analysis of the number of attacks towards a novel stimulus at the nest and the number of captures in a mist net placed at the colony, both measures of neophobia in cliff swallows, in relation to potential life history and environmental predictor variables.

Behavioral measure	Covariate	Post.mean	L CI	U CI	Eff. samp	pMCMC
Number of attacks	Female	0.889	-0.347	2.341	4000	0.125
	Male	0.842	-0.391	2.325	4000	0.138
	Trial rank order 2[a]	-0.073	-0.260	0.107	3873	0.437
	Trial rank order 3[a]	-0.120	-0.338	0.089	4000	0.250
	Trial rank order 4[a]	-0.305	-0.596	0.005	4000	0.046
	Temperature (°C)	-0.090	-0.165	-0.013	4000	0.022
	Wind speed (m/sec)	0.045	-0.032	0.121	4000	0.239
	Extent of sunshine (watts/m^2)	-0.010	-0.083	0.070	4229	0.791
	Days since 1st egg laid	0.008	-0.096	0.110	4000	0.887
	Latency to enter nest	0.158	0.074	0.242	4000	0.0005
Number of captures	Female	0.261	0.126	0.383	2990	< 0.0001
	Male	0.203	0.101	0.305	2972	0.0005

Number of observations: 533; Bird ID and colony Site ID were modelled as a random effects.
$n_{ind.}$ = 160 and n_{sites} = 3.
[a] In relation to trial rank order 1 as baseline.
