# Supplementary material for: Variation in neophobia among cliff swallows at different colonies
Source: PLoS One. 2019 Dec 23;14(12):e0226886. doi: 10.1371/journal.pone.0226886 (PMC6927619; doi:10.1371/journal.pone.0226886)
Supplement: S8 Table — (PDF) [file pone.0226886.s013.pdf]

S8 Table: Bivariate mixed model analysis of latency to enter a nest bearing a novel stimulus, a measure of neophobia in cliff swallows, and reproductive success, measured as the number of nestlings surviving to 17 days of age, in relation to potential life history and environmental predictor variables.

Behavioral measure	Covariate	Post.mean	L CI	U CI	Eff. samp	pMCMC
Latency to enter nest	Female	1.967	-0.854	4.727	4000	0.129
	Male	1.590	-1.326	4.264	4000	0.194
	Trial rank order 2[a]	-1.890	-2.957	-0.869	4000	0.001
	Trial rank order 3[a]	-2.500	-3.610	-1.362	4000	0.0005
	Trial rank order 4[a]	-2.021	-3.688	-0.474	4000	0.012
	Temperature (°C)	-0.148	-0.515	0.240	4000	0.441
	Wind speed (m/sec)	0.051	-0.311	0.419	4000	0.787
	Extent of sunshine (watts/m^2)	0.181	-0.196	0.542	4000	0.343
	Days since 1st egg laid	0.286	-0.247	0.817	3799	0.289
Reproductive success	Lay date	-2.716	-3.502	-2.018	3823	< 0.0001
	Clutch size	0.213	-0.205	0.675	4000	0.358

Number of observations: 533; Bird ID and colony Site ID were modelled as a random effects.
$n_{ind.}$ = 160 and n_{sites} = 3.

[a] In relation to trial rank order 1 as baseline.
