# Supplementary material for: Variation in neophobia among cliff swallows at different colonies
Source: PLoS One. 2019 Dec 23;14(12):e0226886. doi: 10.1371/journal.pone.0226886 (PMC6927619; doi:10.1371/journal.pone.0226886)
Supplement: S9 Table — (PDF) [file pone.0226886.s014.pdf]

S9 Table: Bivariate mixed model analysis of the number of attacks towards a novel stimulus at the nest, a measure of neophobia in cliff swallows, and reproductive success, measured as the number of nestlings surviving to 17 days of age, in relation to potential life history and environmental predictor variables.

Behavioral measure	Covariate	Post.mean	L CI	U CI	Eff. samp	pMCMC
Number of attacks	Female	0.947	-0.484	2.463	4000	0.121
	Male	0.906	-0.455	2.463	4000	0.128
	Trial rank order 2[a]	-0.128	-0.336	0.113	4000	0.267
	Trial rank order 3[a]	-0.171	-0.405	0.078	4000	0.168
	Trial rank order 4[a]	-0.365	-0.705	-0.021	4000	0.036
	Temperature (°C)	-0.091	-0.169	-0.016	4000	0.020
	Wind speed (m/sec)	0.044	-0.028	0.123	4000	0.259
	Extent of sunshine (watts/m^2)	-0.008	-0.088	0.065	4372	0.832
	Days since 1st egg laid	0.002	-0.100	0.101	4352	0.961
	Latency to enter nest	0.159	0.074	0.239	4000	0.0005
Reproductive success	Lay date	-2.672	-3.379	-1.884	3807	< 0.0001
	Clutch size	0.172	-0.265	0.620	4000	0.443

Number of observations: 533; Bird ID and colony Site ID were modelled as a random effects.

$n_{ind.}$ = 160 and n_{sites} = 3.

[a] In relation to trial rank order 1 as baseline.
